# A synthetic population of Sweden: datasets of agents, households, and activity-travel patterns

**DOI:** 10.1016/j.dib.2023.109209

**Published:** 2023-05-07

**Authors:** Çağlar Tozluoğlu, Swapnil Dhamal, Sonia Yeh, Frances Sprei, Yuan Liao, Madhav Marathe, Christopher L. Barrett, Devdatt Dubhashi

**Affiliations:** a Department of Space, Earth and Environment, Chalmers University of Technology, Gothenburg, Sweden; b Department of Computer Science, University of Virginia, Charlottesville, United States; c Department of Computer Science and Engineering, Chalmers University of Technology, Gothenburg, Sweden

**Keywords:** Synthetic population, Activity schedules, Agent-based modelling, Daily activity pattern

## Abstract

A synthetic population is a simplified microscopic representation of an actual population. Statistically representative at the population level, it provides valuable inputs to simulation models (especially agent-based models) in research areas such as transportation, land use, economics, and epidemiology. This article describes the datasets from the Synthetic Sweden Mobility (*SySMo*) model using the state-of-art methodology, including machine learning (ML), iterative proportional fitting (IPF), and probabilistic sampling. The model provides a synthetic replica of over 10 million Swedish individuals (i.e., agents), their household characteristics, and activity-travel plans. This paper briefly explains the methodology for the three datasets: Person, Households, and Activity-travel patterns. Each agent contains socio-demographic attributes, such as age, gender, civil status, residential zone, personal income, car ownership, employment, etc. Each agent also has a household and corresponding attributes such as household size, number of children ≤ 6 years old, etc. These characteristics are the basis for the agents’ daily activity-travel schedule, including type of activity, start-end time, duration, sequence, the location of each activity, and the travel mode between activities.

**Specifications Table**

**Table:** 

Subject	Data Engineering
Specific subject area	Synthetic population with individual activity-travel patterns in Sweden
Type of data	Tables of agents, households, daily activity-travel schedules (.csv data format)
How the data were acquired	We created SySMo model [1] that synthesises the mobility patterns of the entire Swedish population. The model generates three datasets: agents, households, and activity-travel patterns.
Data format	Raw data, Processed (data update, data conversion and data synthesis)
Description of data collection	All input data are obtained by request from the Swedish authorities or downloaded from public sources.
Data source location	Sweden's population statistics [2] Socio-demographic data at Demographic Statistical Areas (DeSO) [3] Total population at square kilometre grids [4] Swedish national travel survey [5] Origin-destination (OD) matrices from Sampers model [6] Building data [7]
Data accessibility	Repository name: Mendeley Data Data identification number: doi:10.17632/9n29p7rmn5 Direct URL to data: http://doi.org/10.17632/9n29p7rmn5

## Value of the Data

• The data, consisting of a synthetic population and households, are crucial inputs to develop simulation models, including agent-based models in various research areas such as transportation, land use, energy, economics, etc.
• The data allows for generating simulations that realistically reflect heterogeneous human mobility behaviour without violating personal privacy.
• The data is created using publicly accessible datasets from Swedish authorities, such as census data and the national travel survey. The methodology can be applied to similar data in other regions.
• The data can be used for models evaluating emerging technologies in transportation in which individual decision-making strongly affects policy outcomes, such as the adoption of electric vehicles or the use of shared autonomous mobility.
• Our datasets enhance research integrity, promote overall transparency, and allow peers to evaluate research outcomes. They provide valuable resources for evidence-based policymaking and scientific research, particularly in the context of national-wide datasets.

## 1 Objective

This paper describes a dataset of Sweden's synthetic population with individual activity-travel patterns and the methodology. Synthetic populations are crucial inputs for developing simulation models, particularly agent-based models, widely used in many research fields, such as transportation, land use, economics, and epidemiology. Although many countries have created synthetic populations (e.g., Switzerland [8], Denmark [9], and Germany [10]), there is a lack of data representing Sweden's entire population to date. The data presented in the paper fills this gap. Synthetic population data has the potential to facilitate research in answering policy-relevant questions that require detailed assessments of potential heterogeneous impacts across the entire population. There are several analytical frameworks that are suitable for utilising data of synthetic populations, depending on the research question and the specific characteristics of the synthetic population. For example: Microsimulation modelling simulates complex systems or processes at the individual level, such as health care, transportation, and labour markets; Agent-based modelling simulates agents' behaviour (individuals or groups) in a given environment, such as social networks, online communities, and marketplaces; Spatial analysis shows the spatial distribution of individuals and groups, such as housing patterns, commuting patterns, and access to amenities to identify spatial inequalities, understand the impact of urban policies, and support urban planning. For instance, using the synthetic population, Liao et al. [11] evaluate the impacts of charging behaviours on battery electric vehicle charging infrastructure in Sweden. Furthermore, making the data open and accessible enhances research integrity in the field, promotes overall transparency in research findings [12]. Open data also provides valuable resources for evidence-based policymaking and scientific research, particularly in the context of national-wide datasets [13].

## 2 Data Description

**Fig. 1:**
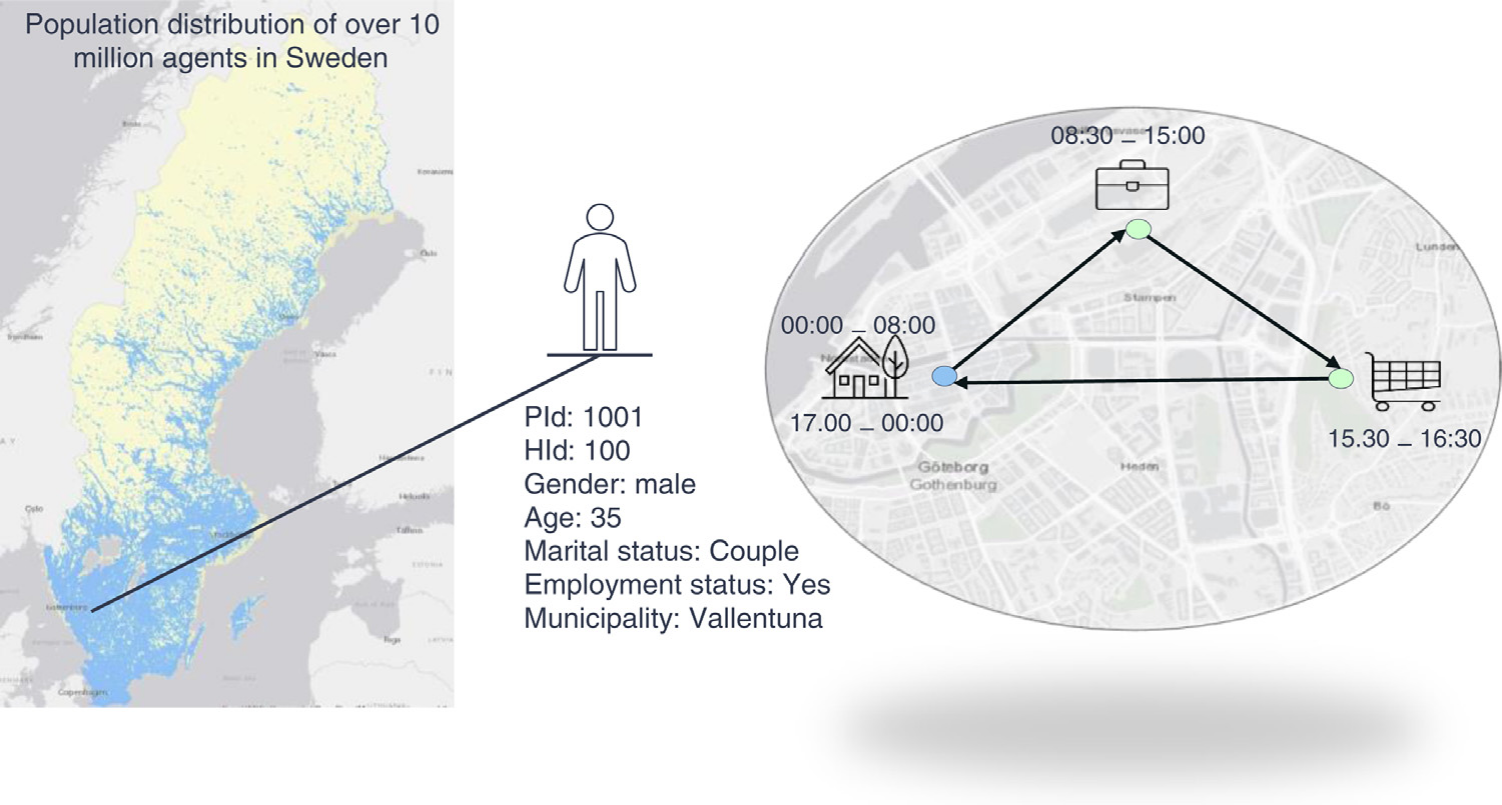
A map of Sweden's residential locations and an illustration of an individual's attributes and daily activities (home-work-other-home).

This paper contains three datasets: Person, Households, and Activity-travel patterns for the year 2018, generated from the Synthetic Sweden Mobility (*SySMo*) model [1]. The datasets are stored in a relational database format in Person, Household, and Activity-travel tables. The Person table (Table 1) contains the synthetic agents representing over 10 million Swedish inhabitants and their socio-demographic attributes. The Household table (Table 3) is formed by individuals in the synthetic population with household features such as household type, size, number of children, and number of cars. The Activity-travel table (Table 5) contains daily activity schedules of agents, i.e., where and when they do certain activities (work, home, school, and other) and how they travel between them (walk, bike, car, and public transport). The tables referenced in this article are available at Mendeley Data [14]. Fig. 1 illustrates an agent with socio-demographic attributes and daily activity-travel pattern.

**Table 1:** Description of the variables in the Person table.

Variable name	Description	Data Type		Share (%)
PId	A unique identifier for an individual (10.203.820 individuals in total)	Integer		-
HId	A unique identifier for the household of an individual (4.653.738 unique households in total)	Integer		-
Gender	Sex	Categorical variable	Male	50.2
			Female	49.8
Age	Age	Integer		-
Marital status	Marital status	Categorical variable	Age < 18	20.5
			Couple	30.9
			Single	48.6
Employment status	Employment status	Categorical variable	Yes	50.0
			No	50.0
Studenthood status	Studenthood status	Categorical variable	Yes	27.4
			No	72.6

Income class	Income class	Categorical variable	No	23.2
			Low	19.0
			Lower-middle	19.4
			Upper-middle	19.2
			High	19.2

Number of cars	Number of vehicles owned by an individual	Categorical variable	0	60.3
			1	37.6
			2	1.7
			3+	0.4

DeSO	Demographic Statistical Areas of the residence (5.985 DeSO in total)	String		-
Municipality	A municipality of the residence (290 municipalities in total)	String		-

The three tables in the dataset are briefly described below with a list of key variables, definitions, and examples.

### 2.1 Person

The Person table contains agents and their attributes, including personal ID, household ID, gender, age, marital status, employment status, studenthood status, income class, number of cars, residential zone, and municipality. Table 1 describes the variables and their statistics, such as the size of the population and the distributions of the categorical variables.

The synthetic population is generated based on data from Statistics Sweden (SCB) [15] at various geographical levels, such as municipality or zone system and the Swedish national travel survey. Each individual is associated with a residential zone called Demographic Statistical Areas (DeSO) [3]. The table shows the employment and studenthood statuses of each individual. While only people over the age of 16 can be employees, people of all ages can be students. Individuals can also have both employment and studenthood status at the same time. Each individual belongs to one of the five income classes (in thousand Swedish krona, kSEK): *I* = {0, [1, 180K), [180K, 300K), [300K, 420K), [420K, 1000K)}. The income classes are based on the Swedish national income quartiles up to 1 million SEK.

Table 2 provides example entries from the Person table.

**Table 2:** Examples from the person table.

PId	HId	Gender	Age	Marital status	Employment status	Studenthood status	Income class	Number of cars	DeSO	Municipality
1	1	1	22	couple	1	0	1	0	0115A0040	Vallentuna
2	1	0	20	couple	0	1	0	0	0115A0040	Vallentuna
3	2	0	20	single	1	1	0	1	0115A0040	Vallentuna
4	3	0	21	couple	1	1	2	1	0115A0040	Vallentuna
5	3	1	20	couple	1	0	4	1	0115A0040	Vallentuna

### 2.2 Household

Table 3 shows the variables of the Household table, including over 4.6 million households with one or more individuals. The household attributes are household ID, type, size, number of children less than six years old, and number of cars in the household. There are three types of households: ’couple,’ ’single,’ and ’other.’ A ’couple’ household consists of a couple with or without children, whereas a ’single’ household consists of an individual with or without children. Any other type of household is named as an ’other’ household (e.g., one with multiple singles or multiple couples or a combination of singles and couples). The variable ’number of children’ accounts for only children less than six years old in households.

**Table 3:** Description of variables in the household table.

Variable name	Description	Data Type		Share (%)
HId	A unique identifier for households	Integer		-
Type	Type of households	Categorical Variable	Single	61.0
			Couple	33.2
			Other	5.8
Size	Number of people living in the households	Integer		-
Number of children	Number of children less than six years old in the household	Integer		-
Number of cars	Number of vehicles in the household	Integer		-

Table 4 shows examples from the Household table.

**Table 4:** Examples from the household table.

HId	Type	Size	Number of children	Number of cars
1	couple	2	0	0
2	single	1	0	1
3	couple	3	1	1

### 2.3 Activity-travel

Table 5 describes the variables of the Activity-travel table. Each individual in the synthetic population has a series of activities on an average weekday. The activity series and the locations of agents are chronologically presented in the Activity-travel table. Since the activity-travel schedules are daily, starting/ending at 3 AM, each individual's first activity of a given day can be considered the continuation of the last activity from the day before. There are four main activities: home, work, school, and others. The travel modes between the activities are car, car passenger, public transport, bike, and walking. Each activity takes place in a specific building whose location and type are assigned based on the building coordinates in the official coordinate system, SWEREF 99 [16]. The three building types are detached houses, apartments, and other buildings. Home can be a detached house or apartment, while work, school, and other activities take place in a building relevant to the activity type.

**Table 5:** Description of variables in the activity-travel table

Variable Name	Description	Data Type	
PId	A unique identifier for individuals	Integer	
Activity ID	A unique identifier showing the individual's activity order	Integer	

Activity Purpose	Purpose of activities	Categorical Variable	Home
			Work
			School
			Other

Activity Start Time	Start time of activities in hours	Float	
Activity End Time	End time of activities in hours	Float	

Travel Mode	Travel mode to access activities	Categorical Variable	Car
			CarPassenger
			PublicTransport
			Bike
			Walking

Building ID	A unique identifier for buildings where activities take place	Integer	

Building Type	Type of buildings	Categorical Variable	Detached house
			Apartment
			Other

Point X	X coordinate of activities	Float	
Point Y	Y coordinate of activities	Float	
DeSO	DeSO code indicating the zone where the activity takes place	String	

Table 6 provides examples from the Activity-travel table.

**Table 6:** Examples extract from the activity-travel table.

PId	Activity ID	Activity Purpose	Activity Start Time	Activity nd Time	Travel Mode	Building ID	Building Type	Point X	Point Y	DeSO
1	0	1	3.0	7.3		4170405	130	684203.6	6617560.5	0115A0040
1	1	4	7.7	13.7	Car P	3725941	399	670042.8	6579363.9	0180C3560
1	2	10	14.2	16.9	Car P	3749926	499	670286.4	6578485.9	0180C3090
1	3	4	17.3	23.3	Car P	3725941	399	670042.8	6579363.9	0180C3560
1	4	1	23.7	3.0	Car P	4170405	130	684203.6	6617560.5	0115A0040
2	0	1	3.0	10.8		4170405	130	684203.6	6617560.5	0115A0040
2	1	10	11.5	19.1	Bike	4153072	699	684282.3	6617753.6	0115A0040
2	2	1	19.8	3.0	Bike	4170405	130	684203.6	6617560.5	0115A0040

**Fig. 2:**
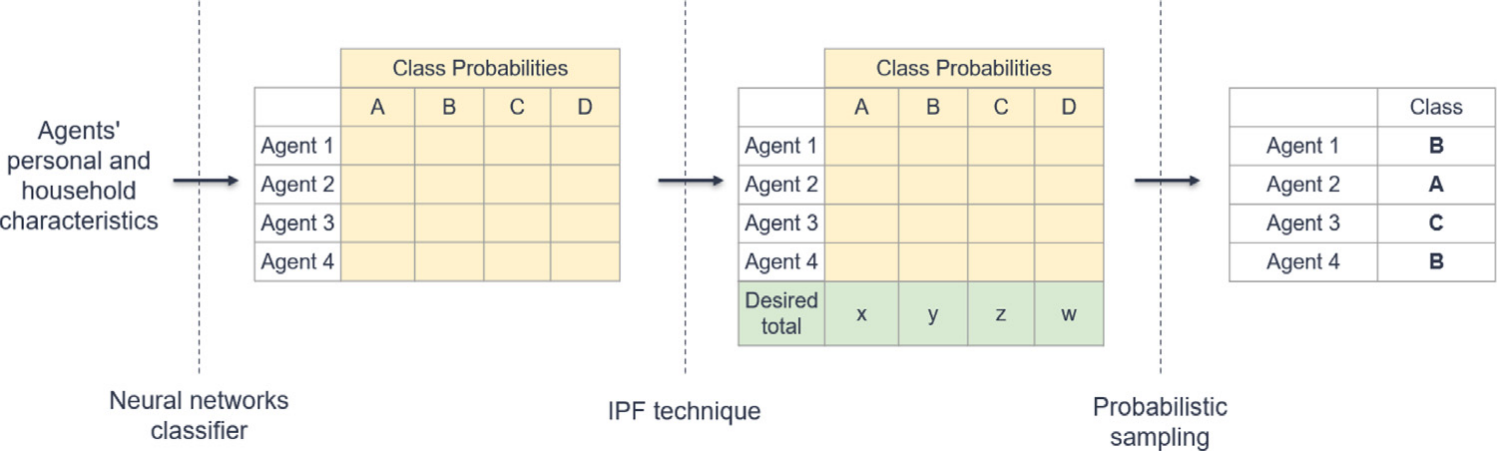
The workflow of the generative probabilistic model. Yellow areas show the joint probabilities for A, B, C, or D choices, such as employment and studenthood statuses. The green area shows each choice's desired total numbers, x, y, z, and w.

**Fig. 3:**
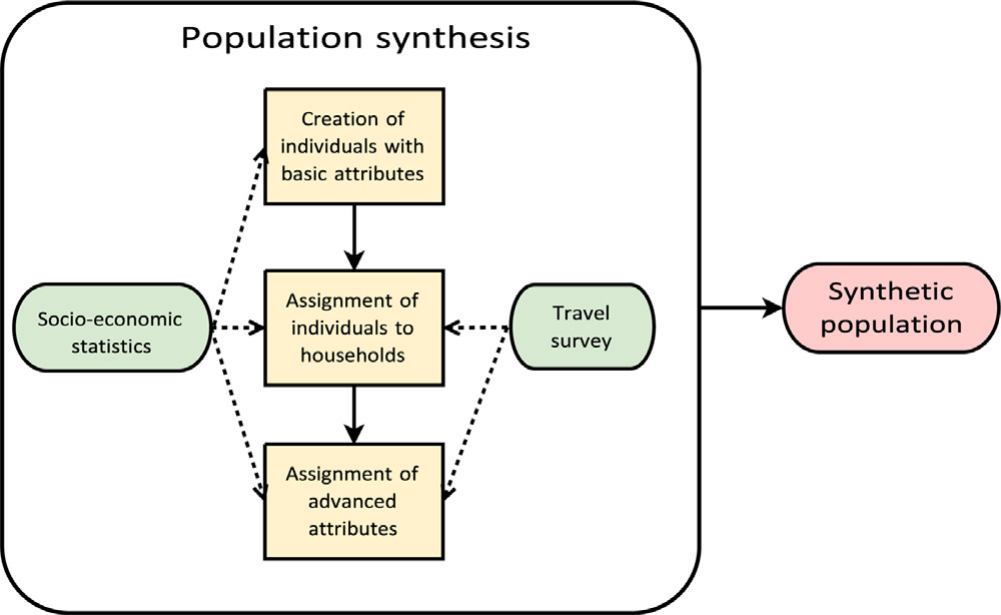
Overview of the population synthesis. Yellow rectangles: major steps of the population synthesis; green ellipses: input data; pink ellipses: outputs of the component.

**Fig. 4:**
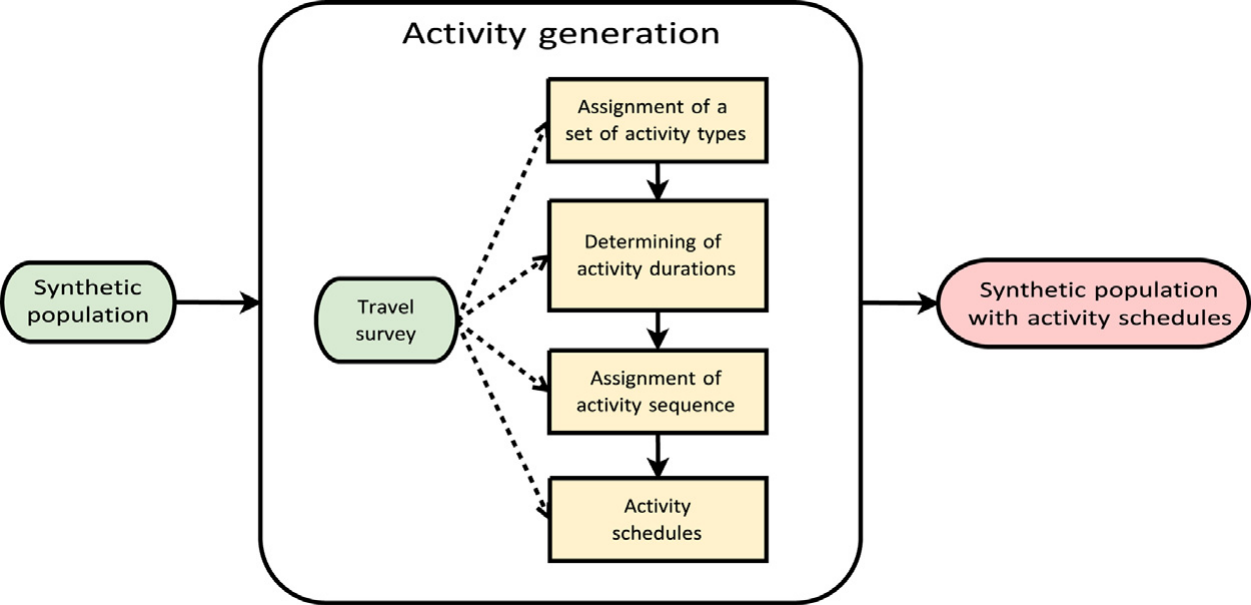
Overview of activity generation. Yellow rectangles: major steps of the activity generation; green ellipses: input data; pink ellipses: outputs of the component.

**Fig. 5:**
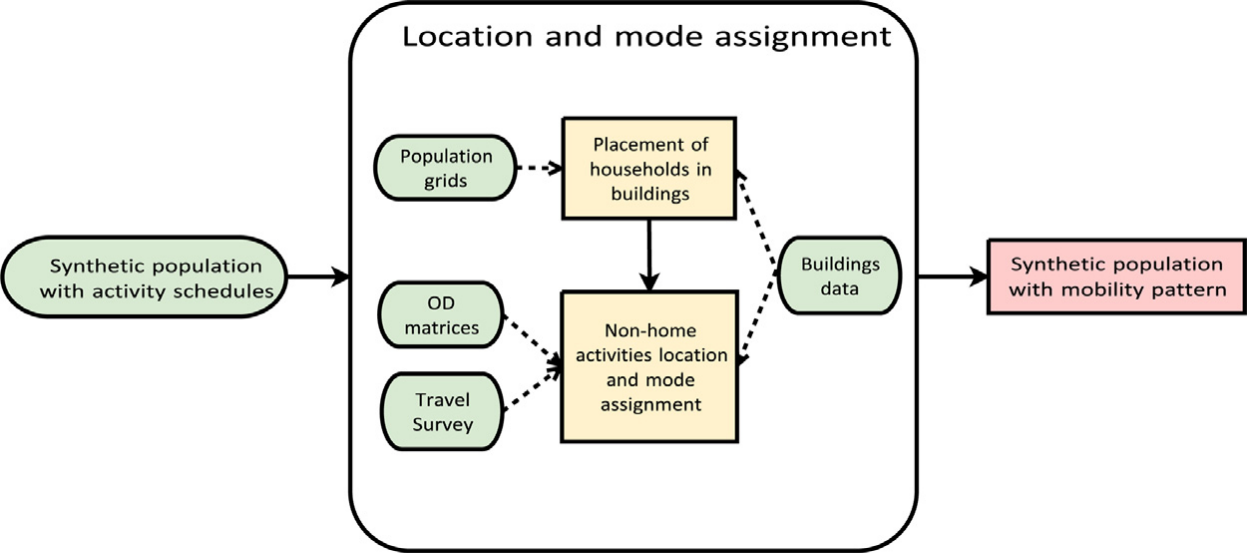
Overview of location and mode assignment. Yellow rectangles: major steps of the location and mode assignment; green ellipses: input data; pink rectangle: the final outputs.

## 3 Experimental Design, Materials and Methods

This section discusses the input datasets and key modelling concepts. The methodology consists of three major steps: 1) population synthesis, 2) activity generation and 3) location and mode assignment. The first component, population synthesis, produces the Person and Household tables. The second and third components produce the Activity-travel table. We also briefly present the assessment and validity of data in this section.

### 3.1 Input data

The creation of a synthetic population of Sweden in this paper uses four primary input sources: statistical data of socio-demographic information from Statistics Sweden (SCB), Swedish national travel survey from Transport Analysis, Origin-Destination (OD) matrices from Trafikverket (Swedish Transport Administration)’s model - Sampers, and buildings from Lantmäteriet (Swedish Land Survey Authority).

#### 3.1.1 Statistical data of socio-demographic information

SCB [15] publishes official statistics at different geographical levels in Sweden, such as the county, municipal or zonal levels. In the population synthesis step, we use municipal data and zone-level statistical data called Demographic Statistical Areas (DeSO) [3]. DeSO zones are formed by dividing municipal boundaries into smaller areas such that each zone consists of a minimum of 700 and a maximum of 2,700 inhabitants. There are a total of 5,984 DeSO zones in Sweden. For each zone, the data contains various socio-demographic variables, i.e., the number of males and females, the number of individuals belonging to different age groups, the number of households of different types (single, couple, other), the number of employees and students, and the number of cars.

To synthesise the population, we also use statistical data at the municipal level [2]. The municipal data consists of the number of individuals with a given combination of gender, age group, and civil status, the number of children belonging to different household types, the number of individuals belonging to different income classes, the average household income of individuals in a given age group belonging to a given household type, and the number of employees by industry types.

SCB also publishes population density data over square kilometre grids. To deduce agents’ residential zones, we use the square kilometre grid data. This grid system presents statistics on the registered population in 114161 square areas covering only populated areas within Sweden.

#### 3.1.2 Swedish national travel survey

Swedish national travel survey [5] is used to obtain employment and studenthood statuses, personal income, and car ownership of the agents and to assign daily activity-travel patterns to the individuals in the population. The Swedish national travel survey presents anonymised individuals’ socio-demographic characteristics, travel habits, and household information. The survey, conducted between 2011 and 2016, contains approximately 40,000 participants aged 6-84.

#### 3.1.3 Origin-destination matrices

The OD matrices from the Swedish transport model, Sampers [6], are used to assign the zones of each activity in the agent's activity plan and to determine the transportation mode between activities. The Sampers model consists of five regional models and a national model. While the national model captures only long-distance trips (more than 100 km), regional models contain information regarding short and long-distance trips. OD matrices are generated by transport modes, i.e., car, bike, walk, public transport, and by trip purposes, i.e., work, business, other, and private. We apply the national and regional models covering the most populated regions with the two largest cities in Sweden: Stockholm and Gothenburg.

#### 3.1.4 Building data

We use the building data, produced from the property registers by Lantmäteriet [7], to determine the precise activity locations within the zone. The data includes information on more than 8.6 million buildings across Sweden. It shows the buildings’ locations, footprint areas, and usage types, i.e., detached houses, apartments, workplaces, schools, and other buildings.

#### 3.1.5 Population synthesis

The agents are generated in three steps (Fig. 3). First, each agent is synthesised along with their basic attributes (e.g. age, gender). Subsequently, households are created using the same attributes. In the last step, the advanced attributes, such as employment status are assigned using a generative model that combines machine learning, iterative proportional fitting, and probabilistic sampling.

We synthesise all agents with the basic attributes, i.e., the age, gender, civil status, residential zone (DeSO), and municipality attributes. The numbers of individuals by gender and age group are available at the DeSO level. At the municipality level, we know the number of individuals with a given combination of gender, age group, and civil status. Using the given statistical data and IPF technique [17], [18], [19], [20], we deduce the joint distribution of age group, gender, and civil status attributes at the DeSO zone level and estimate values in a cross-table of all relevant attributes.

In the household creation step, we create households using synthesised agents’ age and civil status attributes and the data on the number of household types (i.e., ’couple,’ ’single,’ and ’other’) at the DeSO zone level. We begin with the ’couple’ households. For each DeSO zone, we divide individuals with couple marital status by two gender groups. We sort the first group in ascending order of age and the second group in ascending order of an age proxy.^1^ We then match individuals one-to-one based on their order in their group and assign them households. After that, we create ’single’ households by listing the single individuals according to the DeSO-zone level statistics. We then assign children to the created households based on the age difference between the children and the household members. Finally, individuals not assigned to one household are assigned to ’other’ households. Other households can contain multiple singles or couples or a combination of singles and couples individuals.

The last step of the population synthesis is advanced attributes assignment, i.e., employment and student statuses, personal income class, and the number of vehicles for each agent. To deduce these advanced attributes, we use a generative model that combines ML, IPF and probabilistic sampling (Fig. 2). The generative model first uses a neural network classifier (NNC) trained with the Swedish national travel survey. NNC allows for predicting probability distributions of a given set of options (e.g., the probability distribution of an agent belonging to four income classes), while maintaining the correlations between the options in the choice set. Using the IPF techniques, the model subsequently fits the predicted distributions to the desired total numbers, thus ensuring consistency with the official statistics at an aggregated level. Lastly, the generative model generates the agents' choices using random sampling from the probability distributions derived from NNC and fitted by IPF.

We first jointly assign agents employment and studenthood statuses using the generative model, where an NNC is trained on the Swedish national travel survey [5]. We use the basic and household attributes as input to predict the probability distribution of the statuses. We then fit the predicted distributions to the relevant statistics at the DeSO level and randomly sample the employment and studenthood statuses from the learned distribution for each agent. Using a similar procedure, we deduce the personal income class and the number of vehicles.

### 3.2 Activity generation

The activity generation component has four steps to create an activity schedule for each agent (Fig. 4). We start with defining the activity participation of each agent. We then deduce the duration of each activity type in the set and assign an activity sequence. Finally, we create activity schedules for each individual.

For each agent in the synthetic population, we first assign a set of activity types showing the activity participation during the given day. Four types of activities are considered: *home* (*H*), *work* (*W*), *school* (*S*), and *other* (*O*), like visiting shops, restaurants, etc. We assume that each agent visits their home at least once a day. Given the agent's socio-demographic attributes, we use a generative probabilistic model to create a set of activity types, similar to the approach illustrated in Fig. 2.

The activity duration of each activity type is determined through a two-step method. Using another generative probabilistic model, we first jointly deduce broad duration classes (low, moderate, or high) for the different activity types, capturing the correlation between the duration of the different activity types and socio-demographic attributes. We then estimate the total daily travel time for each agent in the form of range classes, $\left(\underline{t_{TT}},\bar{t_{TT}}\right]$ where the lower limit of the range of its daily travel time is $\underline{t_{TT}}$ and the upper limit is $\bar{t_{TT}}$. After assigning the broad travel time class, we determine each activity type's hourly duration. The hourly probability distribution of a given activity type is predicted using an NNC. Then, we sample the duration of all activity types until they collectively satisfy the constraint in Eq. (1).

(1) $$24\;\text{hours}-\bar{t_{\mathrm{TT}}}\leq \;t_{H}+t_{W}+t_{S}+t_{O}\;<24\;\text{hours}-\underline{t_{\mathrm{TT}}}$$

Next, we assign an activity sequence such as home-work-other-home to the agents with known activity types and their durations. An activity sequence comprises the type, order, and frequency of activities participated in a day. To achieve the task, we assume that individuals with similar socio-demographic attributes and activity type duration would have similar activity sequences [21]. We first choose a set of candidate individuals from the travel survey, considering individuals having the same activity participation and having as many similar socio-demographic attributes as possible. To find the most similar individual among candidates, we use the daily activity duration and calculate the Euclidean distance between activity duration tuples (*t_H_,t_W_,t_S_,t_O_*) of individuals. For an agent, the survey participant with the smallest Euclidean distance is selected, and then their sequence is directly assigned to the agent.

After activity types, durations, and sequences are ready, we generate the agents’ activity schedule that contains all their activity instances’ start and end times. We assume the day starts and ends at 3 AM since this is the time of day with the least amount of individuals in motion, according to the travel survey.

We first model the start and end times of the 3 AM activity, which helps arrange the remaining activities using the assigned activity sequences and duration. An NNC is trained to predict 3 AM activity's start and end times. To determine the start and end times of the remaining activities in a sequence (i.e., the activities do not take place at 3 AM), we equally distribute the activity's total duration among its instances in the sequence. For example, if an agent participates in a work activity, followed by other activity types, and then returns to work, the duration of work activities in both instances will be equal.

### 3.3 Location and mode assignment

This component assigns locations to all activities in the daily activity schedules and determines travel modes to access these activities (Fig. 5). First, each household is spatially placed in a residential building classified into a detached house or apartment building. After that, we assign locations to the non-home activities in the schedules and the travel mode (i.e., car as a driver, car passenger, public transit, bike, and walk) between activities.^2^

First, we place households in residential buildings. After the population synthesis (Section 2.2), agents and households have their residences in DeSO zones. To increase the accuracy of the population distribution, we created smaller "virtual zones" by overlapping DeSO and grids of 1 km^2^, from which the size of the population living in the virtual zones are derived. Each household in the DeSO zones is randomly assigned to one of the virtual zones corresponding to the household's DeSO zone. Subsequently, we assign households to detached houses or apartment buildings within each virtual zone by correlating the household size with building type. For example, a larger household is more likely than a single one to live in a detached house.

After assigning home locations, we place non-home activities in the activity sequence and assign the travel mode between the activities. To deduce destination zones and mode, we initially calculate the OD probability matrices for each activity type and travel mode. These matrices provide the probability distribution of an activity located in a zone, given the origin (home) zone, activity type, and travel mode. The OD probability matrices are deduced using OD matrices from the Sampers model or a variant of a gravity model based on the Swedish national travel survey.

We first assign travel modes to agents’ destination locations. Given the OD probability matrices and the agent's socio-demographic attributes, we use a generative probabilistic model to predict modes, similar to the approach illustrated in Fig. 2. Finally, we perform the location assignment in two steps: first zone and then building. We assign activity zones to non-home activities, given the activity type, its origin zone, and the mode used to reach it. Agents having longer travel times in their activity schedules are allocated farther destination zones in the location assignment. At last, all non-home activities are placed in buildings appropriate for their activity type using the building data.

### 3.4 Limitations of the data

While synthetic populations are overall useful for various applications, there are several limitations to consider, such as data availability, bias, lack of transparency, and limitations in predicting behaviours. Accurate and comprehensive data is necessary to create realistic populations; incomplete, inaccurate, or outdated data can limit the model's accuracy. The synthetic population can also be biased if the underlying data is biased. The methodology used to generate the population can be complex, and transparency may be lacking. It may be difficult for researchers to understand how the population was generated and what assumptions were made. Synthetic populations can provide insight into demographic and statistical trends, but they may not accurately predict individual behaviour, especially regarding future technology and behavioural change.

There are certain limitations associated with this dataset. The most important one is the estimation of travel time. Travel durations are estimated based on the time gap between activities. Even though the location assignment for the corresponding activities is performed by correlating travel distances with travel duration, it is not an accurate estimation of the travel time. To better estimate travel durations and the subsequent activities' start-end times, one needs to simulate activity travel plans with an actual road network using a traffic simulation platform such as MATSim [22] and calibrate against empirical traffic data, as implemented in Liao et al. [11].

Because of the limitations listed above, the data should be used with caution and in conjunction with other data sources and analytical methods, and their limitation should be clearly communicated to any potential users.

### 3.5 Evaluation and assessment of the data

We perform data assessments for each model component that generates the data. This subsection provides a brief overview of some of the evaluations. For further details, readers can refer to the model evaluation and assessment chapter in the model documentation [1].

First, we compare the synthesised population against the official statistics at the DeSO zone level and calculate the percentage difference in the number of individuals for each attribute. We found that in over 92% of DeSO regions, the difference in the number of people assigned an incorrect gender by the statistical data is a range between -0.5% to 0.5%. Similarly, the percentage difference for age is within -1% to 1% for more than 78% of DeSO zones. The evaluations show that the attribute distributions of the synthetic population are in good agreement with the statistical data.

**Fig. 6:**
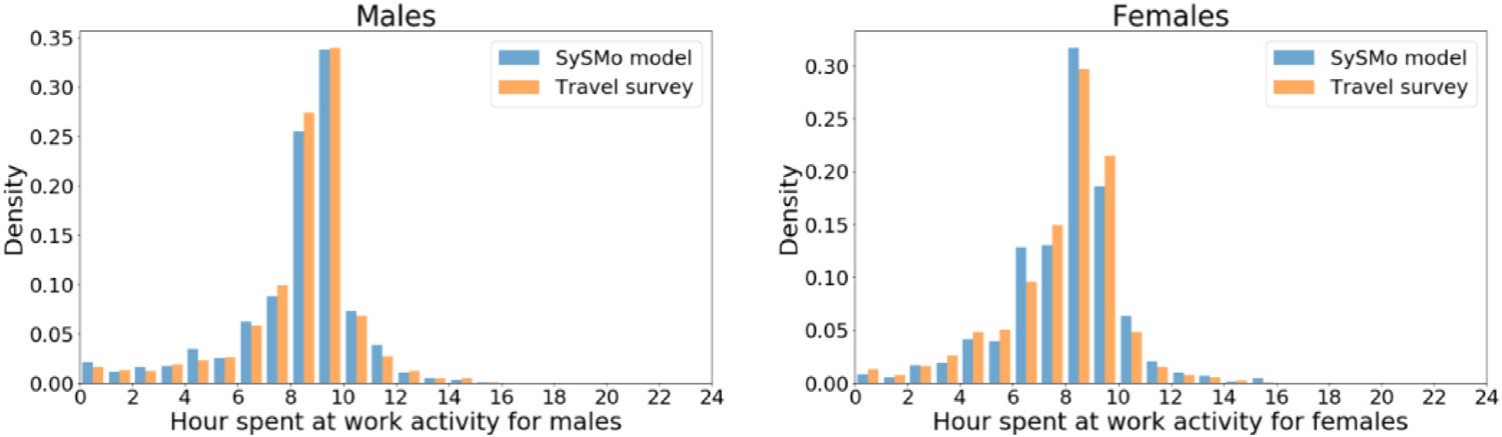
Comparison of work activity duration by gender. The left panel shows the number of hours spent on work activity for males (JS distance=0.05), and the right panel females (JS distance=0.08).

We also compare the generated activity schedules to the travel survey by the activities' duration and temporal profiles and quantify distribution similarity using Jensen-Shannon (JS) distance. JS distances take values in the range from 0 to 1, with 1 indicating the maximum distance. Fig. 6 shows the work activity duration distributions by gender in the data and the travel survey. We calculate JS distance 0.05 for males and 0.08 for females. We repeat comparisons with the school, home, and other activity duration distributions and obtain results in JS distances between 0.05 and 0.13.

**Fig. 7:**
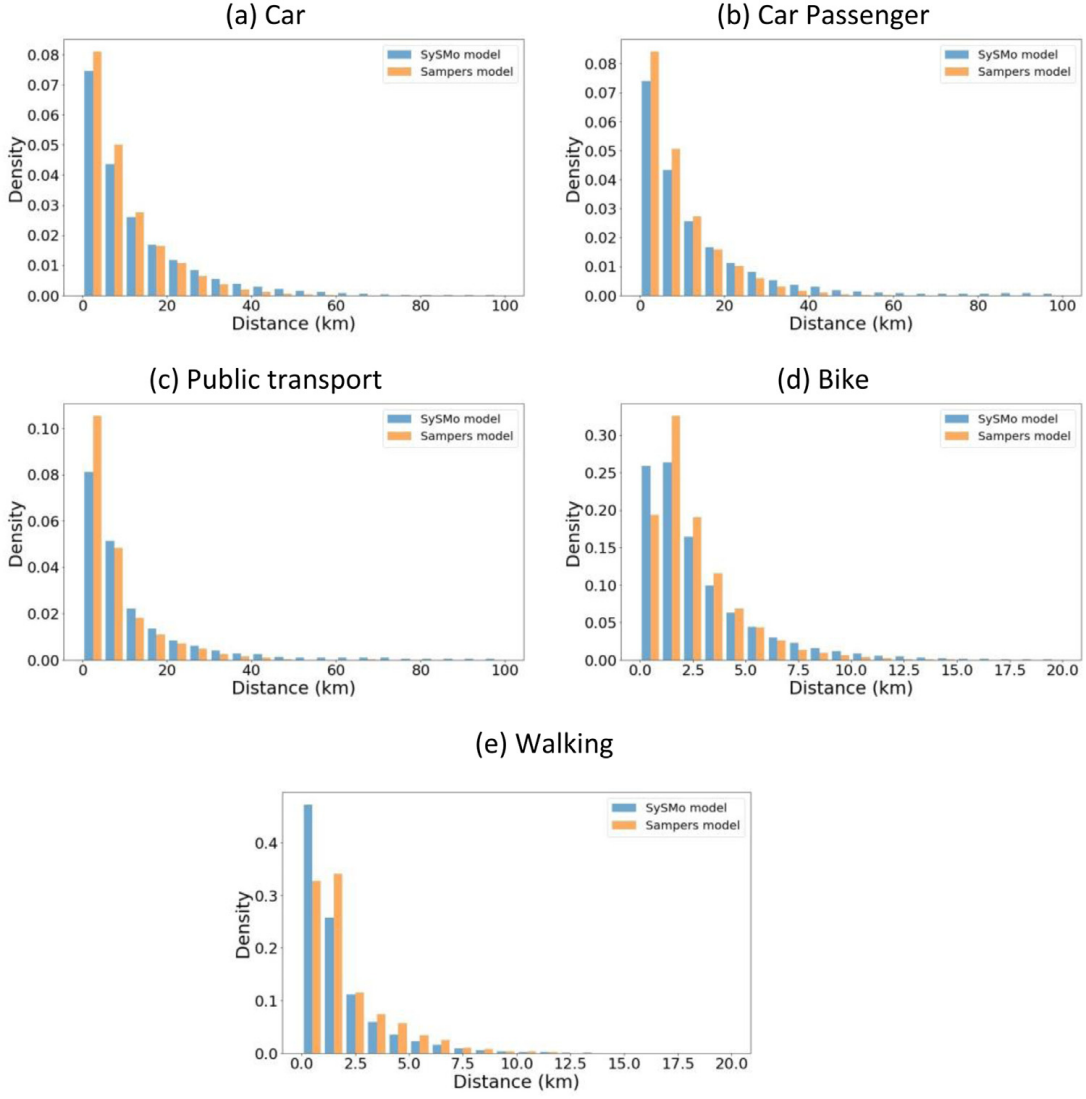
The travel distance distribution between home and work activities by travel modes in Västra Götaland Region.

Finally, we evaluate the performance and validity of synthetic individuals' travel patterns by comparing them with other models, such as the Sampers [6] and one from Trafikanalys [23]. Fig. 7 shows the travel distance distribution between home and work locations by each travel mode in the SySMo and Sampers's west regional model. To compare generated data against the Sampers model, we calculate the spherical travel distance between activity locations by car, car passenger, public transport, bike, and walk. Although there are slight variations in the peak values of the cycling and walking modes, the overall distributions are similar to the Samper model's distributions. The evaluation results show that the activity-travel patterns in the data approximate the validation data patterns reasonably well.

## Ethics Statements

The authors declare that this work does not involve the use of human subjects, social media data, or experimentation with animals.

## CRediT authorship contribution statement

**Çağlar Tozluoğlu:** Conceptualization, Methodology, Software, Validation, Data curation, Writing – original draft, Writing – review & editing. **Swapnil Dhamal:** Conceptualization, Methodology, Software, Validation, Data curation, Writing – original draft, Writing – review & editing. **Sonia Yeh:** Conceptualization, Methodology, Project administration. **Frances Sprei:** Conceptualization, Methodology, Writing – review & editing, Project administration. **Yuan Liao:** Conceptualization, Writing – review & editing. **Madhav Marathe:** Conceptualization. **Christopher L. Barrett:** Conceptualization. **Devdatt Dubhashi:** Conceptualization.

## Declaration of Competing Interest

The authors declare that they have no known competing financial interests or personal relationships that could have appeared to influence the work reported in this paper.

## Data Availability

A synthetic population of Sweden: datasets of agents, households, and activity-travel patterns (Original data) (Mendeley Data).

## Footnotes

1 The age proxy is obtained by sampling a value from a Gaussian distribution for each agent. The agent's age is considered as the mean of the distribution, and the age variance between couples in households in the national travel survey is the standard deviation of the distribution.

2 The non-home activities have two groups, primary (e.g., work and school activities) and secondary (e.g., other) activities. The primary activities’ locations are determined independently. The secondary activities’ locations depend on the location of the primary activities. Please refer to the model documentation [1] for details.
